# Associations between Physical Exercise, Quality of Life, Psychological Symptoms and Treatment Side Effects in Early Breast Cancer

**DOI:** 10.1155/2022/9921575

**Published:** 2022-11-19

**Authors:** Leena Vehmanen, Johanna Mattson, Evangelos Karademas, Albino J. Oliveira-Maia, Berta Sousa, Ruth Pat-Horenczyk, Ketti Mazzocco, Panagiotis Simos, Fátima Cardoso, Greta Pettini, Chiara Marzorati, Eleni Kolokotroni, Georgios Stamatakos, Diana Frasquilho, Paula Poikonen-Saksela

**Affiliations:** ^1^Helsinki University Hospital Comprehensive Cancer Center and University of Helsinki, Helsinki, Finland; ^2^Department of Psychology, University of Crete, Rethymno, Greece; ^3^Foundation for Research and Technology-Hellas, Institute of Computer Science, Heraklion, Greece; ^4^Champalimaud Research & Clinical Centre, Champalimaud Foundation, Lisboa, Portugal; ^5^NOVA Medical School NMS, Universidade Nova de Lisboa, Lisboa, Portugal; ^6^Breast Unit, Champalimaud Clinical Centre/Champalimaud Foundation, Lisboa, Portugal; ^7^School of Social Work and Social Welfare, The Hebrew University of Jerusalem, Jerusalem, Israel; ^8^Applied Research Division for Cognitive and Psychological Science, European Institute of Oncology IRCCS, Milan, Italy; ^9^Department of Oncology and Hemato-oncology, University of Milan, Milan, Italy; ^10^School of Medicine, University of Crete, Rethymno, Greece; ^11^In Silico Oncology and In Silico Medicine Group, Institute of Communication and Computer Systems, School of Electrical and Computer Engineering, National Technical University of Athens, Athens, Greece

## Abstract

**Background:**

Identifying and understanding modifiable factors for the well-being of cancer patients is critical in survivorship research. We studied variables associated with the exercise habits of breast cancer patients and investigated if the achievement of exercise recommendations was associated with enhanced quality of life and/or psychological well-being. *Material and Methods*. 311 women from Finland, Portugal, Israel, and Italy receiving adjuvant therapy for stage I–III breast cancer answered questions about sociodemographic factors and physical exercise. Quality of life was assessed by the EORTC C30 and BR23 questionnaires. Anxiety and depression were evaluated using the HADS scale.

**Results:**

At the beginning of adjuvant therapy and after twelve months, 32% and 26% of participants were physically inactive, 27% and 30% exercised between 30 and 150 minutes per week, while 41% and 45% exercised the recommended 150 minutes or more per week. Relative to other countries, Finnish participants were more likely to be active at baseline and at twelve months (89% vs. 50%, *p* < 0.001 and 87% vs. 64%, *p* < 0.001). Participants with stage I cancer were more likely to be active at twelve months than those with a higher stage (80% vs. 70%,*p* < 0.05). The inactive participants reported more anxiety (*p* < 0.05) and depression (*p* < 0.001), lower global quality of life (*p* < 0.001), and more side effects (*p* < 0.05) than the others at twelve months. Accordingly, those who remained inactive or decreased their level of exercise from baseline to twelve months reported more anxiety (*p* < 0.01) and depression (*p* < 0.001), lower global quality of life (*p* < 0.001), and more side effects (*p* < 0.05) than those with the same or increased level of exercise.

**Conclusion:**

For women with early breast cancer, exercise was associated with a better quality of life, less depression and anxiety, and fewer adverse events of adjuvant therapy. Trial registration number: NCT05095675. Paula Poikonen-Saksela on behalf of Bounce consortium (https://www.bounce-project.eu/).

## 1. Introduction

Breast cancer is the most common malignancy in women and its' prognosis is generally excellent [1]. To facilitate offering interventions and education to promote quality of life and mental health in survivors, it is important to recognize the modifiable factors related to the well-being of these patients. Physical inactivity is a known risk factor for breast cancer [2] and many observational studies suggest an inverse association between physical activity and exercise after breast cancer diagnosis and breast cancer and/or all-cause mortality [3–7]. Exercise interventions [8–15] or physical activity in general [16] have resulted in positive effects on the well-being of breast cancer patients. Small-to-moderate benefits of exercise have been noted concerning the quality of life, physical fitness [8, 11, 12], fatigue [11, 12, 17], and depression [11, 18].

In 2010, the American College of Sports Medicine convened a roundtable to explore the literature on the safety and efficacy of physical exercise both during and after adjuvant cancer therapy and to provide guidelines on appropriate exercise [19]. It was concluded that exercise training is safe both during and after cancer therapies and improves physical functioning, quality of life, and cancer-related fatigue in cancer survivors [19]. The scale of these benefits to physical functioning and quality of life has led to recommending cancer survivors to follow the 2008 US Department of Health and Human Services (US DHHS) Physical Activity Guidelines for Americans [19]. The US DHHS guidelines state that patients with chronic conditions such as cancer should be as physically active as their abilities and conditions allow [20]. Recommended periods of aerobic activity are 150 minutes of moderate-intensity exercise or 75 minutes of vigorous exercise a week. Strength training was recommended 2–3 times per week, along with stretching after each exercise session [19]. These recommendations are in line with the World Health Organization's recommendations on physical activity and sedentary behavior that were updated in 2020 [21]. However, a recent prospective cohort study of US cancer survivors (60% female) showed that physical inactivity is still highly prevalent as only 28% of the survivors reported the recommended 150 minutes or more of leisure time physical activity per week. Moreover, especially a combination of lack of physical activity and prolonged sitting was associated with heightened risks of all-cause and cancer mortality [22].

In this study, we described the exercise habits of early breast cancer patients during the first year from the start of oncological treatments and explored sociodemographic and cancer-related variables that are associated with such factors. Furthermore, we studied whether exercise according to current recommendations had an association with the quality of life or mental well-being of the patients one year after the start of adjuvant therapy.

## 2. Materials and Methods

The BOUNCE project (European Union's Horizon 2020 Research and Innovation Programme under grant agreement No 777167) studies the resilience of early breast cancer patients. Between 2019 and 2020, 816 participants from Finland, Portugal, Israel, and Italy were recruited and gave their consent to be involved in the BOUNCE project. According to the WHO statistics (https://gco.iarc.fr/today/), all the participating countries belong to the category with the highest age-standardized breast cancer incidence worldwide.

Of the 816 participants, 555 were retained in the study after one year. To examine the relationships between aerobic exercise and quality of life and psychological symptoms, we used the data of 311 participants who had completed all the relevant questionnaires at study entry and after one year. Participants with incomplete data were more likely to have less years of education, and to have received chemotherapy or radiotherapy in comparison to those who provided complete data on all variables of interest at baseline and one year later (*p* < 0.01). No other differences in sociodemographic or medical variables between participants with complete and incomplete data were found. The study protocol was approved by the ethics committee of each participating institution and was conducted in accordance with the Declaration of Helsinki. Participants were screened from hospital records, received oral and written information about the study, and provided written informed consent.

### 2.1. Participants

The population of this substudy comprised women aged 40–70 years who were recently diagnosed with histologically confirmed stage I-III breast cancer and received either adjuvant or neo-adjuvant therapy in addition to surgery. Patients received chemotherapy, endocrine therapy, and/or anti-HER2 therapy according to the biological profile of their breast cancer, and radiotherapy according to local guidelines. Patients were eligible if they did not have a history of early onset mental disorder, other serious concomitant diseases diagnosed within the last 12 months, major surgery for severe disease, trauma within 4 weeks prior to study entry, or lack of complete recovery from the effects of surgery or other malignancy within the last five years.

### 2.2. Instruments

We collected participant data regarding sociodemographic factors, lifestyle, and psychological well-being using questionnaires via the online Noona tool or paper. Noona is an electronic patient-reported outcome (ePRO) software for patients with cancer. A trial assistant collected medical information from hospital records. In the BOUNCE project, data was collected from the participants every three months for up to 18 months. In the current substudy, we used data from the baseline and 12 months (M12) after beginning adjuvant therapy.

The participants reported the time, in minutes, spent in aerobic moderate-intensity exercise such as walking, and in vigorous exercise such as running. In accordance with the US DHHS guidelines and for the purpose of our analyses, vigorous exercise was converted to moderate exercise so that one minute of vigorous exercise corresponded to two minutes of moderate exercise. In accordance with a recent British exercise survey (Active Lives Adult Survey May 2020/21 Report, https://sportengland.org/), the patients' physical activity was categorized into three levels: “inactive,” if the amount of exercise was less than 30 minutes a week; “fairly active,” if the amount of weekly exercise ranged from 30 to 149 minutes and “active,” if the amount of weekly exercise was 150 minutes or more.

QOL and adverse effects were measured by the EORTC QLQ-C30 [23] with the breast cancer module supplement BR-23 [24]. The Global health/QOL scale describes patients' subjective quality of life and is based on two questions about physical condition and overall QOL. The Hospital Anxiety and Depression Scale HADS [25] was used to assess the psychological symptoms. Patients with a severe anxiety HADS score of 13 or more at baseline were offered psychological support according to the participating hospital's local practice.

### 2.3. Analyses

To examine the relationships between the level of exercise and sociodemographic and medical factors, we used a series of chi-square tests and ANOVAs. Also, a series of MANOVAs were used to examine the association between the level of exercise at baseline and at one year (M12), as well as the potential change in the level of exercise from baseline to M12 (as the independent variables), and the indices of psychological health and quality of life (i.e., symptoms of anxiety, depression, global quality of life, side effects, arm and breast symptoms; as the dependent variables). These analyses were performed after controlling for several sociodemographic and medical variables. Specifically, we controlled for the country of origin; age; education level (0–9 vs. > 9 years of education); marital status (married/living with a partner vs. single/widowed); employment (employed vs. nonemployed/retired); income (very low/low vs. average/high), as reported by the participants, and after adjusting to the GDP income level of each country; tumor grade; tumor stage (I vs. II and III); therapy type (i.e., lumpectomy vs. mastectomy, radiotherapy, endocrine therapy, chemotherapy, and HER2-directed therapy) coded as a series of dummy variables; body mass index (BMI); receiving vs. not receiving psychotropic medication; receiving vs. not receiving formal psychological support/counselling.

Tests of multicollinearity and multivariate normality (by determining the Mahalanobis distance from the available data and calculating the relevant chi-square values) were performed. Seven outliers were identified, which were excluded from the final sample (i.e., not included in the final sample of 311 participants). A post hoc examination revealed a statistical power of over .90 at an alpha level equal to 5% and a medium effect size, for the analyses performed. The level of statistical significance was defined at *p* < 0.05. The Bonferroni correction for multiple comparisons was used whenever relevant.

## 3. Results

### 3.1. Type of Exercise, Sociodemographic, and Medical Factors

Patient characteristics are presented in Table 1. At baseline, 32% of the participants were inactive (i.e., exercised less than 30 min per week), 27% were fairly active (i.e., exercised 31–149 min per week), and 41% were active (i.e., exercised 150 min or more per week). Twelve months later (M12), 26% of the participants reported being inactive, 30% fairly active, and 45% active. In addition, 36% reported that they stayed inactive or decreased their level of exercise from baseline to M12, whereas 64% reported that they maintained or increased their level of exercise. Of the “inactive” participants at baseline, almost half (49%) exercised more at M12; of the “fairly active” participants at baseline, 22% decreased and 37% increased their level of exercise; of the “active” participants, 34% decreased and 66% maintained their level of exercise. Overall, it was more likely for the participants to maintain or increase their level of exercise than to remain inactive or decrease their level of exercise [64% vs. 36%; *χ*^2^ (4) = 69.97, *p* < 0.001].

Regarding the country of origin and sociodemographic variables (see also Table 2), participants from Finland were more likely to be fairly active or active at baseline (89% vs. 50% on average for all other countries; *χ*^2^ (6) = 67.51, *p* < 0.001). No statistically significant differences existed across education level, marital status, income, tumor stage or grade, type of treatment, and receiving (vs. not receiving) any type of psychotropic medication or formal psychological support at baseline or at M12 (*χ*^2^s < 5.76, *p* > 0.05), as well as BMI and age, (*F*s(2) < 1.90, *p* > 0.05).

Also at M12, participants from Finland were more likely to be fairly active or active (87% vs. 64% on average for all other countries; *χ*^2^ (6) = 24.50, *p* < 0.001). Participants with stage I tumor (vs. stage II or III) were more likely to be fairly active or active at M12 (80% vs. 70%; *χ*^2^ (2) = 6.39, *p* < 0.05). There were no other significant differences regarding all other sociodemographic or medical factors (*χ*^2^s < 6.00, *p* > 0.05), age or BMI, (*F*s(2) < 2.30, *p* > 0.05). Exercise habits at M12 according to sociodemographic and medical variables are presented in Table 2.

Finally, when comparing the change of exercise habits from baseline to M12, the likelihood to maintain or increase the level of exercise was higher in participants from Finland (72% vs. 57% on average for all other countries), with an average or high income (69% vs. 52% for low income), grade I tumor (76% vs. 61% for higher grades), and who were not undergoing chemotherapy (72% vs. 57% for those treated with chemotherapy; *χ*^2^s > 5.90, *p* < 0.05). Also, participants who maintained or increased their level of exercise were older (*M* = 55.34, *SD* = 0.63 years) than those who were inactive or decreased their level of exercise (*M* = 52.96, *SD* = 0.84 years; *F*(1) < 5.13, *p* > 0.05).

### 3.2. Level of Exercise at Baseline and M12 and Health-Related Outcomes

After controlling for sociodemographic (i.e., age, country of origin, education, income, marital status, and employment) and medical factors (i.e., tumor stage and grade, type of treatment, receiving psychotropic treatment or psychological support, and BMI), the level of exercise at baseline was unrelated to any of the outcomes studied, namely psychological well-being, quality of life, side effects, and arm and breast symptoms (Wilks Λ = 0.98, *F*(12, 548) = 0.96, *p* > 0.05, partial *η*^2^ < 0.01). However, the level of exercise at M12 was related to several of the outcomes (Wilks Λ = 0.89, *F*(12, 548) = 2.79, *p* < 0.001, partial *η*^2^ = 0.06; see also Table 3). The effects of the level of exercise at M12 remained statistically significant even after controlling for baseline well-being and quality of life indices (Wilks Λ = 0.90, *F*(12, 536) = 2.45, *p* < 0.01, partial *η*^2^ = 0.05). Specifically, compared to active participants, inactive participants reported more symptoms of anxiety (*F*(2, 273) = 3.85, *p* < 0.05, partial *η*^2^ = 0.03) and depression (*F*(2, 273) = 9.49, *p* < 0.001, partial *η*^2^ = 0.07), lower global quality of life (*F*(2, 273) = 7.31, *p* < 0.001, partial *η*^2^ = 0.05) and more side effects (*F*(2, 273) = 4.57, *p* < 0.05, partial *η*^2^ = 0.03). According to the pairwise comparisons, no statistically significant difference existed between fairly active and active participants (see also Table 3). Regarding arm and breast symptoms, no statistically significant differences were found (*F*s(2, 273) < 0.35, *p* > 0.05, partial *η*^2^s < 0.01).

The change in the level of exercise from baseline to M12 was also related to several of the outcomes at M12 (Wilks Λ = 0.91, *F*(6, 275) = 4.41, *p* < 0.001, partial *η*^2^ = 0.09; see also Table 4). These effects remained significant even after controlling for baseline well-being and quality of life indices (Wilks Λ = 0.90, *F*(6, 269) = 4.99, *p* < 0.001, partial *η*^2^ = 0.10). Specifically, those who remained inactive or decreased their level of exercise reported more symptoms of anxiety (*F*(1, 274) = 7.32, *p* < 0.01, partial *η*^2^ = 0.03) and depression (*F*(1, 274) = 25.30, *p* < 0.001, partial *η*^2^ = 0.09), lower global quality of life (*F*(1, 274) = 13.92, *p* < 0.001, partial *η*^2^ = 0.05) as well as more side effects (*F*(1, 274) = 4.95, *p* < 0.05, partial *η*^2^ = .02; see also Table 4). There were no significant differences regarding arm and breast symptoms (*F*s(1, 274) < 0.45, *p* > 0.05, partial, *η*^2^s < 0.01).

## 4. Discussion

Our findings showed that women with early breast cancer were more likely to maintain or increase their level of exercise than to stay inactive or decrease their level of exercise over one year from the start of oncological treatments (64% vs. 36%; *χ*^2^ (4) = 69.97, *p* < 0.001). This is in line with findings of the largest prospective exercise intervention study on breast cancer survivors BREX, whereby physical activity increased both in patients randomized to exercise intervention and control groups during the early rehabilitation period [26]. However, other evidence suggests that women with early breast cancer significantly decrease their physical activity and exercise levels from one-year prediagnosis to one-year postdiagnosis when types of sports and household activity were assessed with an interview [27]. It is important to consider that the BOUNCE study period overlapped with the beginning of the COVID-19 pandemic era which could have had an impact on the results. According to a recent review, however, physical activity levels decreased, and sedentary behavior increased during the COVID-19 lockdown across various populations [28] while breast cancer survivors in our study managed to maintain their activity levels. Former exercise habits predict exercise behavior during cancer survivorship [29], which was also true in our study as a significant correlation was found between exercise activity at baseline and after one year (Spearman rho = 0.54, *p* < 0.001). Despite the participants being more likely to maintain or increase than to decrease their activity level, only 45% of them did aerobic exercise according to the given recommendations after one year. This fact emphasizes the need for patient education to promote their physical activity from the start of oncological treatment. Patient education could help breast cancer patients to prevent maladaptive lifestyle behaviors, such as weight gain or sedentariness, and lower the risk of cancer relapse or even death [30].

In the current study, the Finnish participants were physically more active than participants from Portugal, Israel, and Italy. This is in line with the statistics published by the European Commission in 2017, which reported that the highest proportions of people in the EU who spent at least 150 minutes per week exercising lived in Finland (54.1%) (https://ec.europa.eu/eurostat/web/products-eurostat-news/-/DDN-20170302-1). Treatment type had no significant impact on exercise activity one year after the beginning of treatment. This lack of significance pertains to the relation between the most acute adverse events and chemotherapy or radiotherapy, which at one year have already been completed and the adverse events have eased. However, stage I patients were more active after one year than stage II and III participants. The exercise habits of study participants with more advanced stages of cancer may be affected by other factors.

Inactivity at twelve months was significantly related to several outcomes, namely to more symptoms of anxiety and depression, lower global quality of life, and more side effects. According to the available literature, exercise intervention has positive effects on several of these outcomes [31]. The positive effects of exercise mainly apply to exercising according to the recommendations of at least 150 minutes of aerobic exercise per week divided into 2–3 times [31]. Our study suggests that also exercising for 30–150 minutes per week, or increasing the amount of exercise may also have positive effects on the quality of life and mental health. The effect size of the impact of the exercise level (inactive, 30–149 minutes, 150 minutes, or more) on the outcomes after one year was medium to small. However, the effect size of the change in exercise level was medium to high (especially regarding depressive symptoms), indicating that a stable level of moderate or high exercise or an increased level of exercise activity is significantly related to better well-being. Thus, motivating patients to exercise according to general recommendations is also pertinent due to other demonstrated health benefits [20]. Accordingly, people are able to transfer skills (e.g., a person-centered perspective) from sports to other life domains, such as oncological health management. The positive attitude and behaviors learned during sports activities may be transferred to other life contexts, helping patients in coping with mixed feelings, such as uncertainty and fear of recurrence [32].

The strength of the present study is that its participants represent a typical breast cancer population receiving various breast cancer treatments while coming from different cultural backgrounds. In the context of the BOUNCE project, extensive background material was available from a breast cancer cohort who received standard treatment, without any additional exercise intervention that could lead to the selection of more active patients for the study [26]. The literature about the physical activity of breast cancer patients concerns mainly randomized trials with some type of exercise intervention [9, 10, 12–15, 33], which can also affect patients' activity in the control group [26]. One limitation of our study is that measuring physical activity was based on participants' self-reports, without any objective digital activity measurements. Another limitation is the suboptimal sample size with a large number of drop-outs and thus a possibility of a selection bias. The study was prospective, yet correlational; therefore, no causal effects can be inferred.

## 5. Conclusions

Exercise after diagnosis of breast cancer was associated with a better quality of life, less depression, and fewer adverse events of adjuvant therapy. However, one year after beginning adjuvant therapy, less than half (45%) of the women diagnosed with breast cancer exercised according to current guidelines. Promoting motivation for physical activity among breast cancer patients from the time of diagnosis throughout the treatment phase and the first-year postdiagnosis is vital for improving their mental health and quality of life.

## Figures and Tables

**Table 1:** Patient characteristics *n* = 311.

Variable	Mean	Range
Age, years	55.41 (SD = 7.99)	40–70
BMI, kg/m^2^	26.05 (SD = 4.45)	17.26–40.89
ECOG	% at baseline	% at M12
0	90.8%	71.1%
1	8.9%	24%
2	0.3%	4.6%
3	—	0.3%
Country	N	%
Finland	143	46%
Portugal	76	24.4%
Israel	53	17%
Italy	39	12.5%
Education		
0–9 years	14	4.5%
>9 years	297	95.5%
Marital status		
Married/living with a partner	272	87.5%
Single/widowed	39	12.5%
Working		
Employed	230	74%
Nonemployed/retired	81	26%
Income		
Very low/low	74	23.8%
Average/high	237	76.2%
Tumor grade		
I	53	17%
II	152	48.9%
III	106	34.1%
Stage		
I	147	47.3%
II	136	43.7%
III	28	9%
Operation		
Lumpectomy	231	74.3%
Mastectomy	80	25.7%
Radiotherapy		
Yes	247	79.4%
No	64	20.6%
Endocrine treatment		
Yes	263	84.6%
No	48	15.4%
Chemotherapy		
Yes	170	54.7%
No	141	45.3%
Anti-HER2 treatment		
Yes	57	18.3%
No	254	81.7%
Psychotropic medication		
Yes	84	27%
No	227	73%
Psychological support		
Yes	36	11.6%
No	275	88.4%

BMI = body mass index, ECOG = performance status, M12 = after twelve months from the start.

**Table 2:** Exercise habits at month 12 according to sociodemographic and medical variables (Ν = 311).

Variable		Inactive (*n* = 80)	Fairly active (*n* = 92)	Active (*n* = 139)
Age, years		*M* = 53.99 (SD = 7.39)	*M* = 55.79 (SD = 7.91)	*M* = 55.94 (SD = 8.33)
BMI, kg/m2	≤25	29 (20.6%)	42 (29.8%)	70 (49.6%)
	>25	51 (30%)	50 (29.4%)	69 (40.6%)
Country				
Finland		19 (13.3%)	46 (32.2%)	78 (54.5%)
Portugal		24 (31.6%)	21 (27.6%)	31 (40.8%)
Israel		20 (37.7%)	15 (28.3%)	18 (34%)
Italy		17 (43.6%)	10 (25.6%)	12 (30.8%)
Education				
0–9 years		9 (64.3%)	2 (14.3%)	3 (21.4%)
>9 years		71 (23.7%)	90 (30.4%)	136 (45.9%)
Marital status				
Married/living with a partner		70 (25.7%)	75 (27.6%)	127 (46.7%)
Single/widowed		10 (25.6%)	17 (43.6%)	12 (30.8%)
Working				
Employed		61 (26.5%)	66 (28.7%)	103 (44.8%)
Nonemployed/retired		19 (23.5%)	26 (32.1%)	36 (44.4%)
Income				
Very low/low		27 (36.5%)	19 (25.7%)	28 (37.8%)
Average/high		51 (21.5%)	73 (30.8%)	113 (47.7%)
Tumor grade				
I		9 (17%)	17 (32.1%)	27 (50.9%)
II		39 (25.7%)	42 (27.6%)	71 (46.7%)
III		32 (30.2%)	33 (31.1%)	41 (38.7%)
Stage				
I		30 (20.4%)	41 (27.9%)	76 (51.7%)
II & III		50 (30.5%)	51 (31.1%)	63 (38.4%)
Operation				
Lumpectomy		55 (23.8%)	72 (31.2%)	104 (45%)
Mastectomy		25 (31.3%)	20 (25%)	35 (43.8%)
Radiotherapy				
Yes		58 (23.5%)	78 (31.6%)	111 (44.9%)
No		22 (34.4%)	14 (21.9%)	28 (43.8%)
Endocrine treatment				
Yes		63 (24%)	76 (28.9%)	124 (47.1%)
No		17 (35,4%)	16 (33.3%)	15 (31.3%)
Chemotherapy				
Yes		50 (29.4%)	51 (30%)	69 (40.6%)
No		30 (21.3%)	41 (29.1%)	70 (49.6%)
Anti-HER2 treatment				
Yes		11 (19.3%)	19 (33.3%)	27 (47.4%)
No		69 (27.2%)	73 (28.7%)	112 (44.1%)
Psychotropic medication				
Yes		21 (25%)	25 (29.8%)	38 (45.2%)
No		59 (26%)	67 (29.5%)	101 (44.5%)
Systematic psychological support				
Yes		9 (25%)	14 (38.9%)	13 (36.1%)
No		71 (25.8%)	78 (28.4%)	126 (45.8%)

BMI = body mass index.

**Table 3:** The means and standard deviations (in parenthesis) of psychological well-being and quality of life indices across participants' level of exercise at month twelve (*n* = 311). Only the indices with a statistically significant difference across the various levels of exercise are included in the Table.

Index	Inactive *n* = 80 (25.7%)	Fairly active *n* = 92 (29.6%)	Active *n* = 139 (44.7%)
Anxiety	0.93 (0.65)	0.65 (0.42)	0.71 (0.52)
Depression	0.80 (0.66)	0.46 (0.39)	0.41 (0.40)
Global quality of life	66.77 (22.44)	77.54 (15.61)	78.84 (16.58)
Adverse events	22.56 (17.41)	15.27 (11.61)	18.36 (13.28)

**Table 4:** The means and standard deviations (in parenthesis) of psychological well-being and quality of life indices across the change in the level of exercise from baseline to month 12 (*n* = 311). Only the indices with a statistically significant difference across the changes in levels of exercise are included in the Table.

Index	Inactivity or decreased exercise *n* = 112 (36%)	Same or increased exercise *n* = 199 (64%)
Anxiety	0.87 (0.05)	0.68 (0.49)
Depression	0.71 (0.60)	0.42 (0.40)
Global quality of life	69.64 (21.13)	78.56 (16.31)
Adverse events	20.92 (16.04)	17.18 (12.96)

## Data Availability

The data that support the findings of this study are available upon request from the corresponding author PPS. The data are not publicly available due to restrictions e.g., they are containing information that could compromise the privacy of research participants.
